# Treatment of Central Vertigo With Low Dose Olanzapine: Report of Two Cases

**DOI:** 10.7759/cureus.22647

**Published:** 2022-02-27

**Authors:** Connie Jiang, Anusha Lekshminarayanan, Ihsan Balkaya, Alal Uddin, Sheital Bavishi, Eric Altschuler

**Affiliations:** 1 Physical Medicine and Rehabilitation, Private Practice, Fairfax, USA; 2 Physical Medicine and Rehabilitation, Metropolitan Hospital Center, New York, USA; 3 Physical Medicine and Rehabilitation, The Ohio State University College of Medicine, Columbus, USA

**Keywords:** olanzapine, treatment, peripheral, central, vertigo

## Abstract

Good treatments are available for many cases of vertigo due to a peripheral cause such as benign paroxysmal positional vertigo. Conversely, vertigo secondary to a central lesion remains a treatment challenge typically without good pharmacologic or other treatments. We have successfully treated two patients, the first to our knowledge, with central vertigo, one from brain injury, one after stroke, with low dose olanzapine which we found to quickly and dramatically resolve vertigo and permit functional normalization. In our two cases, we found that a low dose of olanzapine 2.5mg daily (typical dosing of olanzapine for the psychiatric disease is 5-20mg daily) caused vertigo to rapidly and dramatically remit. Interestingly, our two cases had different causes and possibly lesion locations.

## Introduction

Vertigo can have peripheral and central causes [1]. The most important differentiating facts are peripheral vertigo presents with predominant vestibulocochlear signs and symptoms of vertigo, tinnitus, and/or hearing impairment, whereas central vertigo is often associated with other brainstem signs and symptoms. Central vertigo is a condition in which an individual experiences hallucinations of motion of their surroundings, or a sensation of spinning, while remaining still, as a result of dysfunction of the vestibular structures in the central nervous system (CNS) [2]. 

Central vertigo can occur when there is a lesion or dysfunction of the brainstem vestibular apparatus - vestibular nuclei and their projections, especially to the cerebellum [3]. Central vertigo is clinically characterized by purely vertical spontaneous nystagmus, purely torsional with direction change on lateral gaze, or downbeating. It can last for weeks to months and usually has little effect with head position changes, but this can occur.

Vertigo secondary to a stroke or brain injury can be symptomatically and functionally disabling and has no consensus treatment [4]. Olanzapine is an atypical antipsychotic approved for the treatment of schizophrenia and bipolar depression. Olanzapine was for central vertigo based on its effectiveness as an anti-emetic [5]. Here we report the successful treatment of two cases of central vertigo with a low dose of olanzapine. 

Cases one and two were presented at the 2018 and 2021 American Academy of Physical Medicine and Rehabilitation Annual Assembly, respectively.

## Case presentation

Case 1

**Figure 1:**
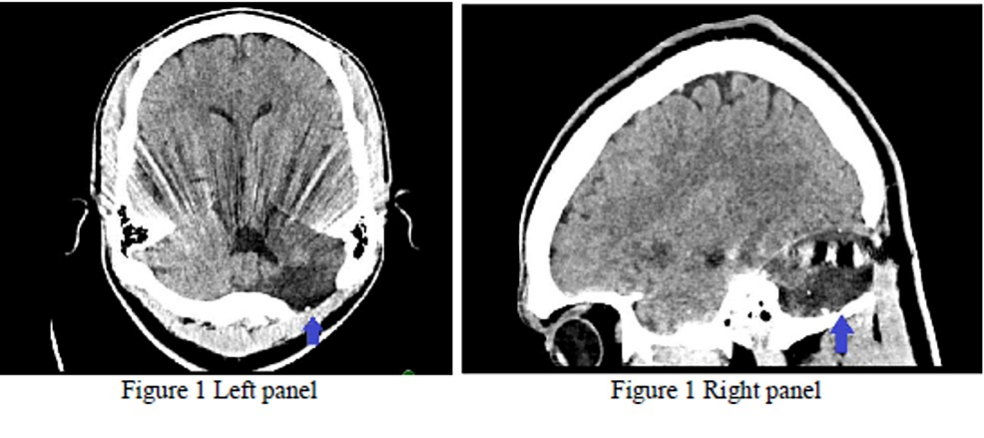
CT of the first patient Left panel: axial view CT shows bullet trajectory and left cerebellar hemisphere (arrow) encephalomalacia, streak artifact from a retained bullet fragment. Right panel: sagittal view CT shows bullet trajectory through left cerebellar hemisphere (arrow) with associated encephalomalacia.

The first case is a 20-year-old male who sustained a gunshot wound to the head. The initial Glasgow Coma Scale was 14, with the unknown duration of loss of consciousness. The bullet entered the left ear, traversed the inferior portion of the left cerebellar hemisphere, and terminated in the midline occipital region. Other significant findings included a subdural hematoma along with the posterior foramen magnum. He underwent a left suboccipital craniotomy for posterior fossa decompression (Figure 1). Otorhinolaryngology performed a left mastoid wound exploration with no significant findings: surgical evaluation of mastoid entry wound noted no cerebral spinal fluid leak and washout, mastoid was intact as well as external auditory canal. He developed anxiety and nightmares, which were treated with olanzapine for seven days. 

Upon admission to inpatient rehabilitation eight days after injury, he had symptoms of vertigo that did not interfere with his function. Symptoms of vertigo included loss of balance, room spinning with turning in bed, and delay in focus with head-turning. His anxiety and nightmares had resolved, so olanzapine was discontinued after a total of seven days of treatment. Within two days of this medication discontinuation, his vertigo worsened. Physical therapy (PT) performed testing, including head thrust test, which was positive for corrective saccades bilaterally, head-shaking nystagmus test, which was positive for dizziness and negative for nystagmus, roll test, which was positive bilaterally left greater than right, and left Dix-Hallpike which was positive for dizziness and vertical nystagmus. This testing revealed motion sensitivity and impaired vestibular-ocular reflex (VOR) consistent with central vestibular hypofunction. From this and the clinical history, it was concluded that the patient's vertigo was of central origin. The patient's symptoms developed into intractable nausea and vomiting, which did not respond to ondansetron, prochlorperazine, scopolamine patch, or meclizine. He could not tolerate therapies or even a clear liquid diet. He had three days of medical misses for therapy due to dizziness, nausea, and vomiting. After more than a week of trialing different medications, olanzapine 2.5mg daily was started, given its off-use label for nausea in cancer patients [5]. The patient's vertigo, nausea, and vomiting improved after one day of treatment. After three days, his symptoms were 95% completely resolved. We do not believe that olanzapine was having an anxiolytic effect because he still was able to sleep, did not have nightmares, and participated in other therapies such as speech therapy, rehabilitation psychology, and occupational therapy (OT) as long as all activities were bed level. After three months, his vertigo had resolved, and olanzapine was discontinued.

Case 2

**Figure 2:**
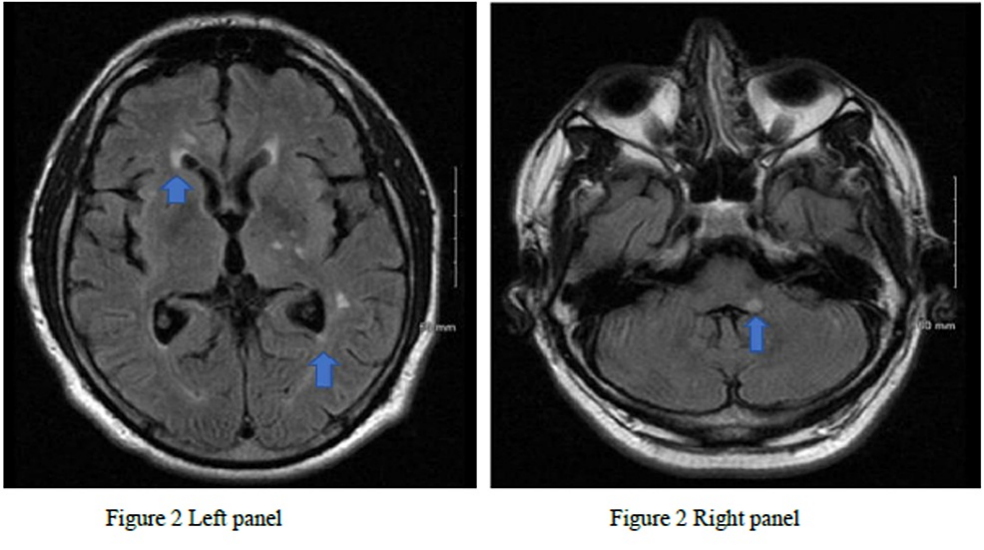
MRI of the second patient Left panel: T1 weighted images of the second patient showed acute infarcts in the right periventricular white matter (arrow). Right panel: T1 weighted images of the second patient showed acute infarcts in the left medulla (arrow).

A 53-year-old male presented with severe dizziness, nausea, vomiting, lower limb weakness leading to a fall with a brief loss of consciousness. Initial CT head and cervical spine were negative. Lumbar X-rays showed bilateral minimally displaced sacral wing fractures. Orthopedics recommended weight bearing as tolerated for the right leg and toe-touch weight-bearing for the left leg. The patient's vertigo did not improve with IV hydration and meclizine. MRI of the head revealed an acute infarct in the left medulla and right periventricular white matter (Figure 2) and chronic infarcts in the cerebellum, brainstem, corona radiata, thalamus, and basal ganglia.

In acute rehabilitation, his strength was 5/5 on the right side and 4/5 on the left. On initial PT and OT evaluations and for two more days, the patient refused to attempt standing due to vertigo. As in the first case, testing by physical therapy was consistent with central vestibular dysfunction. This, combined with the history and imaging, again led us to conclude that the second patient also had vertigo of central origin. On day 3 of acute inpatient rehabilitation (AIRD3), meclizine 50mg orally twice daily was initiated along with olanzapine 2.5mg oral daily based on its anti-emetic effect [5]. Vertigo improved significantly. The next day, he ambulated 20'; on AIRD7, he performed stair training. At discharge (AIRD18), he was asymptomatic, ambulated 350' and 12 stairs. Low-dose olanzapine and meclizine were extremely effective in alleviating our patient's symptoms and leading to significant functional gains. We think olanzapine was responsible for the resolution of the patient's vertigo as previously, meclizine alone had not been at all beneficial for vertigo. On a one-month follow-up, the patient's vertigo had resolved, and olanzapine and meclizine were discontinued.

## Discussion

There are good treatments for some peripheral and central causes of vertigo [1]. For example, benign paroxysmal positional vertigo, which is caused by calcite crystals that have been dislodged from the utricle and are moving freely in the semicircular canals, can often be successfully treated by physical exam crystal repositioning maneuvers [6,7]. Vertigo, secondary to many kinds of strokes and brain injuries, remains a treatment challenge without consensus [4]. The cases discussed above are the first in the literature to demonstrate that low-dose olanzapine could help resolve central vertigo and restore quality of life. Indeed, in a study of prescription patterns and off-label use of antipsychotics (first- and second-generation), Bastaki et al. [8] found that while antipsychotics are not uncommonly used to treat benign positional vertigo, there is no mention of off-label use of these drugs for central vertigo. 

We decided to use olanzapine for our patients' central vertigo, given its effectiveness as an anti-emetic [5]. Olanzapine was also found effective in a treatment-resistant case of severe nausea and vomiting in a patient with brain metastases from a rectal carcinoma [9]. When taken for long periods, olanzapine can cause weight gain and type II diabetes [10]. Also, with long-term use, there is a risk of extrapyramidal motor symptoms [11]. When starting olanzapine, patients need to be apprised of these risks. Our two cases had different etiologies-brain injury and stroke, so olanzapine may be generally useful for central vertigo, but the mechanism of action of olanzapine on vertigo requires future study.

## Conclusions

Low dose olanzapine was effective in alleviating vertigo in two patients with brain lesions: one from a stroke, the other secondary to brain injury. Future studies of olanzapine for vertigo secondary to stroke or brain injury may be warranted. 
